# Metaverse in dentistry: Bridging virtual innovation with real-world patient care and education

**DOI:** 10.6026/973206300210262

**Published:** 2025-02-28

**Authors:** Anchal Deep Kaur Brar, Pallavi K.M, Maneesha Das, Prabhat Saxena, Kaustubh Kulkarani, Prerna Priya, Ritik Kashwani

**Affiliations:** 1 Department of Prosthodontics and Crown & Bridge, Government Dental College, Silchar, Assam, India; 2 Department of Dental Sciences, Sharda University, Greater Noida, Uttar Pradesh, India; 3 Department of Conservative Dentistry and Endodontics, Institute of Dental Sciences, Siksha O Anusandhan University, Bhubaneswar, Odisha, India; 4 Department of Conservative Dentistry and Endodontics, ITS Dental College, Muradnagar, Ghaziabad, Uttar Pradesh, India; 5 Department of Oral and Maxillofacial Surgery, Bharati Vidyapeeth (Deemed to be University) Dental College and Hospital, Pune, Maharashtra, India; 6 Department of Conservative Dentistry and Endodontics, AIIMS Rishikesh, Uttarakhand, India; 7 Department of Oral Medicine and Maxillofacial Radiology, School of Dental Sciences, Sharda University, Greater Noida, Uttar Pradesh, India

**Keywords:** Artificial Intelligence, dental education, extended reality, metaverse, patient care

## Abstract

Integration of metaverse technology in dentistry is transforming patient treatment, education and professional growth. This investigation examines the awareness, attitude and preparation of dental students and practitioners regarding metaverse applications in their field of work. Hence, 476 participants were asked about their awareness of artificial intelligence, XR, block-chain and digital twins, together with the various advantages and disadvantages of these technologies. While metaverse adoption is widely sought, especially in education and training, questions about cost, privacy and usability still linger. Thus, the need for structured learning programs to maximize the benefits of metaverse technology and raise dental practitioners' digital proficiency is highlighted.

## Background:

Among the disciplines most likely to be disrupted digitally, dentistry has revolutionized healthcare most radically with technology [1]. Dental patient treatment, education and professional training can be changed by extended reality (XR), artificial intelligence (AI), block-chain, cloud computing and metaverse, an immersive digital environment [2]. Virtual simulations, AI-driven diagnostics and tele-dentistry applications represent among the main innovations that can increase access, accuracy and participation in dental practice [3]. The potential to create interactive and immersive learning environments, offer remote consultations and allow precision-driven dental surgeries utilizing digital twins and AI-based analytics shows the immense opportunities of this technology [4, 5].

Though metaverse technologies in dentistry offer significant benefits, knowledge of and acceptance of them remain rare [6]. Many students and dental professionals do not know enough about the applications, challenges and moral concerns around adding the metaverse into ordinary surgeries [7]. Knowing the degree of knowledge and preparedness among dental students and professionals would help one estimate the future relevance of this technology in dentistry [8, 9]. Therefore, it is of interest to assess the knowledge, opinion and preparedness of 476 dental students and professionals to incorporate metaverse technology into their clinical and academic operations.

## Methodology:

476 participants, including undergraduate and postgraduate dental students, interns and faculty members, were used in a cross-sectional research to evaluate their awareness, perspective and readiness to apply metaverse technology in dentistry. Using a systematic questionnaire, the study covered important areas like demographic information, understanding of metaverse applications, possible advantages in patient care, education and training, as well as obstacles to adoption using WhatsApp groups and email. Using frequency distribution, chi-square analysis and logistic regression, the gathered answers were examined to find trends, patterns and correlations between several demographic groupings. This analytical technique clarified how several elements, including education degree, experience and technology familiarity, affect opinions and preparedness to accept the metaverse in dental practice.

## Results:

The study involved 476 people overall-graduates and undergraduate dentistry students, interns and faculty members among other things. The bulk of respondents, 50.8%, were between the ages of 21 and 25; followed by those between the ages of 20 and younger (39.3%) and a lesser number aged 26 or older (9.9%) (Figure 1 - see PDF) Comparatively to male participants (27.1%), the research included more female participants (72.9%). The participants' educational background showed that 62.8% were undergraduate students, while the balance included professors (3.2%), postgraduates (18.7%) and interns (15.3%). The academic and professional backgrounds of people using metaverse technologies in dentistry are thoroughly presented in this demographic study (Figure 2 - see PDF).

## Knowledge:

Although dentistry students, interns, postgraduates and faculty members have a modest degree of knowledge of met averse technologies, the results of this study highlight very significant knowledge gaps. Reflecting a modest familiarity with the idea, a notable 57.9% of participants claimed having heard of the met averse. Still, the awareness of certain met averse-related technology differed greatly. With 76.9% of respondents saying they know about artificial intelligence (AI) applications in dentistry, especially in areas like diagnostics and treatment planning, this technology became the most often known one. 52.3% of the participants also agreed that Extended Reality (XR), which includes augmented reality (AR), virtual reality (VR) and augmented virtuality (AV), has promise in offering immersive learning opportunities and thereby enhancing patient care. On the other hand, understanding of Blockchain (11.5%), Internet of Things (IoT) (13.2%) and Digital Twinning (17.3%), was much lower, suggesting that these technologies are either less known or more opaque in the framework of dental practice. This difference in knowledge emphasises the necessity of further educational initiatives to include all facets of met averse technology in the dialogue within the dentistry community. Although many of the participants did not completely understand their particular uses or advantages in the dentistry sector, notwithstanding a large number of them were aware of the potential that the met averse technologies may provide. This suggests an opportunity for further research and teaching on these tools.

## Attitude:

Evaluation of participants' opinions on the possible integration of met averse technologies into dentistry found generally favourable attitudes, especially in the fields of education, patient care and training. Most participants indicated excitement about using met adverse technologies in their professional lives as they understood the possibility of virtual consultations and immersive learning environments to improve patient involvement and education. Many dental professionals and students are open to using these cutting-edge technologies as most (84.9%) indicated they would be ready to include met averse applications in clinical settings. Particularly for patient education and oral health promotion, participants thought met averse technologies were rather helpful; 89.6% of them agreed that these technologies would greatly improve patient knowledge of dental operations and support improved oral hygiene practices. Furthermore, 59% of respondents said met averse might help with diagnostic and treatment planning as immersive tools provide the possibility for more exact and customised therapy. Notwithstanding this excitement, participants also underlined various adoption difficulties, mostly related to metaverse technology (60.7%), privacy and security issues (53.6%) and usability problems (41.4%). These obstacles show that, although met averse technologies are much supported, real obstacles-especially technical and financial ones-must be removed before general adoption can take place. Overall, the attitude evaluation unequivocally shows hope for the future of met averse in dentistry, balanced with a pragmatic awareness of the challenges that have to be solved.

## Practice:

The study revealed a noteworthy desire among participants to apply met adverse technology in their regular practice in the field of dentistry. For patient education and oral health promotion especially, a substantial majority (84.9%) of participants were amenable to including met averse in clinical practice. Most participants felt that, particularly with virtual consultations and interactive learning opportunities, met averse might help to increase patient access to dental treatment. In dentistry training, where 90.2% of participants were willing to employ met averse technologies for skills development, notably hands-on learning via virtual simulations and haptic technology, this tendency was very clear. These instruments provide trainees with a special chance to practice difficult surgeries in a risk-free, controlled setting. Moreover, especially by offering immersive, realistic training experiences, 91.2% of participants thought that met averse would greatly improve students' abilities and confidence. Though most participants expressed a strong desire to adopt met averse, they also pointed out the pragmatic obstacles stopping complete adoption. The most often mentioned difficulty was the expense of integrating met averse technology; 60.7% of respondents said that many practices would find the financial outlay needed to embrace these technologies unacceptable. Significant hurdles to adoption also came from privacy issues (53.6%) and usability problems (41.4%). These results imply that even if the dental community is excited about the possibilities of met averse technologies, significant efforts are required to solve pragmatic problems, especially regarding financial support, organised training programs and the development of user-friendly interfaces (Table 1).

## Discussion:

Kalaimani *et al.* [10] highlighted important gaps in reason, particularly technologies despite the general consciousness of nascent digital tools. While Kalaimani's study shows 63.5% awareness of AI among participants, our study reflects a higher awareness of 76.9% specifically regarding AI Uses in diagnostics and treatment was planning. Notwithstanding both studies, it is necessary to take further targeted informative initiatives to work these gaps and better the reason for particular uses in dentistry regarding attitudes both studies break bold perspectives along integration of artificial intelligence into dental do. Most participants in both studies showed their forecast about AI's potential to improve dental care and educational opportunities. Similarly, both studies spotlight the development concern in incorporating artificial intelligence into the program to check that dental professionals are spread to employ these technologies when it comes to doing our information. However, both studies also suggest that practical use and hands-on encounters remain areas that need further development. Dzyuba *et al.* study [11] regarding practice, both studies share the perspective that hands-on learning and immersive training tools, such as VR simulations or Metaverse applications, can significantly enhance clinical education and patient interactions. There is a shared belief that these technologies offer a way to bridge gaps in traditional training, especially in complex procedures where risk-free practice is essential. Zhu *et al.* [12] study and our data show a strong positive attitude toward the integration of digital technologies in dentistry, including AI, Metaverse and XR tools. Both studies find that there is high enthusiasm for incorporating new technologies into clinical practice and education. This is reflected in the high percentage of participants in both studies expressing willingness to adopt these tools, especially for improving patient care, education and training. Our study highlighted the potential of Metaverse technologies, particularly VR and XR, in dental education, enhancing training through immersive learning environments and hands-on experiences. Chauhan *et al.* [13] also emphasized the role of tele-dentistry and AI-driven diagnostics in improving patient care, with virtual consultations helping to reduce dental anxiety and personalize patient experiences. However, both studies identify significant knowledge gaps about technologies like blockchain, AI and XR, stressing the need for structured training. Cost and privacy concerns are major barriers to adoption in both clinical and educational settings. Lastly, both studies call for further research, policy development and financial support to integrate these technologies into dental curricula and expand professional training.

## Challenges, opportunities and the path forward:

From patient care improvements to practical training opportunities, the metaverse is ready to transform dentistry in many different ways. To acquaint students with XR, blockchain and AI-driven diagnoses, however, successful integration of digital literacy programs into the dentistry curriculum will call for Subsidies and financial support to help remove cost obstacles and advance accessibility. Investing in training programs helps to solve the lack of knowledge and improve usability; developing ethical rules and regulatory systems guarantees patient safety and data protection. The future of this technology depends critically on longitudinal research evaluating the efficacy of metaverse-based treatments in real-world dental practice and education. Although metaverse integration generated great excitement, participants pointed out various obstacles and issues such as high cost of technology and equipment (60.7%), which makes it difficult for institutions and private practices to commit to metaverse-based infrastructure. Privacy and cyber-security issues (53.6%), which arise when employing metaverse platforms, raise questions regarding data security and patient confidentiality. Usability and accessibility issues (41.4%), therefore stressing the importance of user-friendly interfaces and reasonably priced solutions to guarantee general adoption. Lack of understanding in using metaverse apps (37.4%), pointing to a knowledge gap that has to be filled by organized training courses. Lack of metaverse rules in dentistry (27.7%) among participants for stressing the necessity of legal frameworks and consistent procedures for ethical and safe use is important. Ethical questions including patient's autonomy, informed consent and the ethical consequences of virtual therapies arising from ethical issues in metaverse-driven therapy (39.1%). These results highlight the need to tackle these issues to guarantee a seamless shift toward metaverse adoption in dentistry education and clinical activity.

## Conclusion:

With enormous enthusiasm among students and professionals, especially in education, training and patient care, the metaverse seems to have significant promise to transform dentistry. Successful adoption depends on addressing issues including cost, privacy and knowledge gaps. The dentistry sector may use the metaverse to improve patient outcomes and professional training with correct education, policy creation and financial assistance.

## Figures and Tables

**Table 1:** KAP assessment of the study

**Category**	**Knowledge (K)**	**Attitude (A)**	**Practice (P)**
**General Awareness**			
Have you heard of Metaverse previously?	Yes: 276 participants (57.9%) / No: 200 participants (42.1%)	Positive interest in learning about Metaverse.	15.1% (50 participants) are not willing to incorporate Metaverse into clinical practice.
**Are you aware of any of the following technologies?**			
Extended Reality (AR, VR, AV)	Yes: 249 participants (52.3%) / No: 227 participants (47.7%)	Enthusiastic about the potential use of XR technologies in education and patient care.	62% (295 participants) were willing to use XR technologies in their practice.
Blockchain	Yes: 55 participants (11.5%) / No: 421 participants (88.5%)	Scepticism about the role of blockchain due to limited understanding.	63.5% (302 participants) were willing to incorporate blockchain into their practice.
Internet of Things (IoT)	Yes: 63 participants (13.2%) / No: 413 participants (86.8%)	Interest in IoT applications to improve practice management and patient care.	13.4% (63 participants) have practised using IoT in their professional environment.
Artificial Intelligence	Yes: 366 participants (76.9%) / No: 110 participants (23.1%)	High enthusiasm for AI-driven diagnostics and treatment planning tools.	85.7% (408 participants) believe AI can improve surgical accuracy and precision.
**Metaverse in Dentistry**			
Are you aware of the use of Metaverse in Dentistry?	Yes: 98 participants (20.6%) / No: 378 participants (79.4%)	Positive view towards adopting Metaverse, especially in education and patient care.	20.7% (98 participants) are aware of its use in dentistry.
Can Metaverse be useful in patient care?	Yes: 278 participants (58.5%) / No: 198 participants (41.5%)	Strong belief in its potential for interactive patient education and reducing anxiety.	62% (295 participants) think Metaverse can provide a personalized service experience.
Can Metaverse be used for patient education?	Yes: 268 participants (56.4%) / No: 208 participants (43.6%)	Strong belief in the potential of Metaverse to educate patients on their dental health.	89.6% (294 participants) are willing to incorporate Metaverse for patient education.
Can Metaverse improve treatment planning and diagnosis?	Yes: 281 participants (59%) / No: 195 participants (41%)	Positive views on its potential to improve diagnostic accuracy and efficiency.	59.1% (182 participants) believe Metaverse could improve treatment planning and diagnosis.
**Metaverse in Patient Education**			
Oral health promotion	Yes: 426 participants (89.5%) / No: 50 participants (10.5%)	High enthusiasm for Metaverse applications to improve oral health awareness.	89.6% (294 participants) were willing to use Metaverse for oral health promotion.
Reduction of dental anxiety	Yes: 410 participants (86%) / No: 66 participants (14%)	Many participants agreed that Metaverse could help reduce patient anxiety during procedures.	33.8% (104 participants) have used Metaverse for anxiety reduction in patient care.
Developing oral hygiene habits	Yes: 414 participants (87%) / No: 62 participants (13%)	Most agree that Metaverse could motivate patients to improve oral hygiene.	80.5% (264 participants) see the potential for Metaverse gamification in promoting good dental habits.
Understanding the treatment procedure	Yes: 294 participants (89.6%) / No: 182 participants (10.4%)	Strong belief in Metaverse helping patients visualize and understand the treatment process.	89.6% (294 participants) see Metaverse as a tool to improve understanding of the treatment procedure.
**Metaverse in Dental Training**			
Are you aware of the use of Metaverse for dental training?	Yes: 312 participants (65.5%) / No: 164 participants (34.5%)	Strong enthusiasm for integrating Metaverse tools into dental training programs.	66% (315 participants) see value in Metaverse providing realistic experiences for students.
Hands-on learning opportunities	Yes: 243 participants (51%) / No: 233 participants (49%)	Positive view on how Metaverse can enhance hands-on learning experiences.	50.7% (243 participants) see hands-on learning as one of the primary benefits of Metaverse.
Haptic technology for manual dexterity	Yes: 191 participants (40.1%) / No: 285 participants (59.9%)	Interest in learning more about using haptic technology to refine manual skills.	43.1% (206 participants) want to practice with haptic technology for manual skill development.
Virtual simulations for dental surgeries	Yes: 220 participants (46.2%) / No: 256 participants (53.8%)	Positive view on the benefits of simulated surgeries for educational purposes.	46.1% (220 participants) support using virtual simulations for dental surgeries.
Will Metaverse increase skills and confidence?	Yes: 299 participants (91.2%) / No: 35 participants (8.8%)	Great enthusiasm towards using Metaverse for skill development and boosting confidence.	91.2% (299 participants) believe it will enhance students' skills and confidence.
**Metaverse in Practice**			
Willingness to incorporate Metaverse into clinical practice	Yes: 404 participants (84.9%) / No: 72 participants (15.1%)	Positive attitude towards its adoption in clinical practice, especially for education and patient care.	84.8% (404 participants) are open to incorporating Metaverse into clinical practice.
**Challenges and Barriers**			
Cost of implementing Metaverse technology	Yes: 289 participants (60.7%) / No: 187 participants (39.3%)	Concerns about the cost of technology adoption in dental practice.	60.7% (289 participants) think cost is a significant barrier to Metaverse adoption.
Privacy and security concerns	Yes: 255 participants (53.6%) / No: 221 participants (46.4%)	Privacy and data security are major concerns when it comes to using Metaverse platforms in dental practice.	53.6% (255 participants) consider privacy to be a major concern.
Usability and accessibility issues	Yes: 197 participants (41.4%) / No: 279 participants (58.6%)	There is a need for user-friendly interfaces and affordable solutions for widespread adoption.	41.4% (197 participants) mentioned accessibility and usability challenges as key concerns for Metaverse adoption.
**Blockchain for Record-keeping**			
Are you aware of Blockchain in dental record keeping?	Yes: 185 participants (38.9%) / No: 291 participants (61.1%)	Interest in the potential for blockchain to secure and streamline dental records.	63.4% (302 participants) support the integration of blockchain technology for record keeping.
**Advantages of Blockchain**			
Ease in record keeping and maintenance.	Yes: 128 participants (62.4%) / No: 48 participants (37.6%)	High recognition of blockchain's potential in simplifying data management.	62.4% (128 participants) see blockchain as a solution for improving record keeping.
Data protection	Yes: 107 participants (52.2%) / No: 169 participants (47.8%)	Significant concern about ensuring data privacy and protection in digital records.	52.2% (107 participants) are aware of blockchain's potential for protecting data.
**Additional Questions**			
How likely are you to recommend Metaverse applications to your colleagues?	Yes: 357 participants (75%) / No: 119 participants (25%)	The majority would recommend Metaverse applications to colleagues for improving education and patient care.	40% (190 participants) have already recommended Metaverse-based tools to their peers.
Do you believe Metaverse can increase patient access to dental care?	Yes: 346 participants (72.7%) / No: 130 participants (27.3%)	High support for improving patient access to dental services through virtual platforms like Metaverse.	31.8% (151 participants) have used virtual consultations to improve access to care.
How often do you use digital tools or software in your daily practice?	Yes: 245 participants (51.5%) / No: 231 participants (48.5%)	80% (380 participants) view the integration of Metaverse into digital tools as a natural progression.	51.5% (245 participants) frequently use digital tools in their practice, which could include Metaverse applications.
Do you think Metaverse can be used to reduce the skill gap in dental education?	Yes: 299 participants (62.9%) / No: 177 participants (37.1%)	The majority believe Metaverse can help bridge the skills gap by providing immersive, interactive learning.	66% (314 participants) feel that Metaverse could help reduce the skills gap in dental education.
Would you support funding or research into Metaverse applications for dentistry?	Yes: 391 participants (82%) / No: 85 participants (18%)	High support for funding and research to explore further applications of Metaverse in the dental field.	82% (391 participants) would support funding research into Metaverse applications for dentistry.
